# Biochemical analysis of CTLA-4 immunoreactive material from human blood

**DOI:** 10.1186/1471-2172-10-51

**Published:** 2009-09-22

**Authors:** Matt Tector, Bhupendra O Khatri, Karen Kozinski, Kate Dennert, Martin K Oaks

**Affiliations:** 1 Aurora St Luke's Medical Center, Transplant Research Lab, 2900 W Oklahoma Ave, Milwaukee, WI 53215 USA; 2 Center for Neurological Disorders, SC, #630 2801 W Kinnickinnic River Pkwy, Milwaukee, WI 53215 USA

## Abstract

**Background:**

CTLA-4 was initially described as a membrane-bound molecule that inhibited lymphocyte activation by interacting with B7.1 and B7.2 molecules on antigen presenting cells. Alternative splicing of mRNA encoding the CTLA-4 receptor leads to the production of a molecule (sCTLA-4) that lacks a membrane anchor and is therefore secreted into the extracellular space. Despite studies finding that people with autoimmune disease more frequently express high levels of sCTLA-4 in their blood than apparently healthy people, the significance of these findings is unclear.

**Methods:**

Molecules isolated from blood using CTLA-4 specific antibodies were analyzed with ligand binding assays, mass spectroscopy, and biochemical fractionation in an effort to increase our understanding of CTLA-4 immunoreactive material.

**Results:**

Mass spectroscopy analysis of the molecules recognized by multiple CTLA-4-specific antibodies failed to identify any CTLA-4 protein. Even though these molecules bind to the CTLA-4 receptors B7.1 and B7.2, they also exhibit properties common to immunoglobulins.

**Conclusion:**

We have identified molecules in blood that are recognized by CTLA-4 specific antibodies but also exhibit properties of immunoglobulins. Our data indicates that what has been called sCTLA-4 is not a direct product of the CTLA-4 gene, and that the CTLA-4 protein is not part of this molecule. These results may explain why the relationship of sCTLA-4 to immune system activity has been difficult to elucidate.

## Background

Alternate splicing of the CTLA-4 mRNA transcript can give rise to at least three mRNA species that encode different polypeptides [1]. The most well characterized of these is a type I transmembrane protein (CTLA-4-TM) expressed on activated T-lymphocytes [2,3]. CTLA-4-TM is a co-receptor for the B7.1 (CD80) and B7.2 (CD86) molecules expressed on antigen presenting cells [4,5]. CTLA-4-TM inhibits immune activity in multiple ways. It regulates signaling through the T-cell receptor [6,7], induces expression of immunoregulatory factors such as TGF-β and ICAM-1 [8,9], alters the organization of the immunological synapse [10], increases tryptophan catabolism by antigen presenting cells [11,12] and binds B7.1 and B7.2 preventing activation of lymphocytes through the costimulatory lymphocyte receptor CD28 [13]. Another transcript of the CTLA-4 gene encodes a molecule lacking the transmembrane domain, thus producing a soluble CTLA-4 polypeptide referred to as sCTLA-4 [14,15]. Like CTLA-4-TM, sCTLA-4 appears to bind B7.1 and B7.2, and may have immunomodulatory properties [15-17]. Finally, a variant transcript [18] has been identified in mouse (although not humans) that encodes a membrane-spanning molecule with an intact cytoplasmic tail, but lacks the extracellular domain. As such, the molecule does not bind the B7 family ligands [19] and has been referred to as ligand-independent CTLA-4 (liCTLA-4).

The expression of these three CTLA-4 transcripts and their polypeptide products has been associated with immunoregulatory function, and differences in their expression have been associated with immune-mediated disease. For example, CTLA-4 knockout mice express none of the possible alternate transcripts and show a profound lymphoproliferative disorder with fatal multiorgan destruction [20,21]. Although it is commonly believed that the absence of the CTLA-4-TM transcript is solely responsible for the observed immunological disorder in CTLA-4-knockout mice, the role(s) of the other transcripts have not been studied as intensively. LiCTLA-4 may have immunoregulatory functions, as transfection of it into CTLA-4 deficient T cells partially corrects the tendency for hyperresonsiveness [19], and the liCTLA-4 transcript has been associated with the development of insulin dependent diabetes mellitus in the NOD mouse [18]. Finally, a variety of reports implicate a role for sCTLA-4 in human autoimmune disease. The CT60 single nucleotide polymorphism of the CTLA-4 gene has been associated with autoimmunity and with reduced levels of the sCTLA-4 transcript [18]. Various studies have demonstrated elevated levels of the sCTLA-4 protein in the blood of patients with a variety of immunologically mediated diseases including autoimmune thyroid disease [22], systemic lupus erythematosis [23,24], cutaneous systemic sclerosis [25], allergic asthma [26,27], and psoriasis vulgaris [28]. This apparent inverse relationship between levels of sCTLA-4 mRNA and circulating levels of the sCTLA-4 protein is not understood.

Several years ago, we [22] and others [14] described immunoassays for the detection of sCTLA-4 in human plasma. Presumably, such material was the gene product of the sCTLA-4 transcript; however, this was never formally proven. In order to characterize sCTLA-4 in human blood, we performed biochemical analyses of blood-derived molecules that are recognized by multiple CTLA-4-specific antibodies. Our results suggest that the immunoreactive material in human blood is not the direct product of the sCTLA-4 alternate transcript and has several biochemical features of human immunoglobulin. In addition, CTLA-4 immunoreactive material from human plasma binds the B7.1 and B7.2 proteins, and may have immunomodulatory function.

## Methods

### Monoclonal Antibodies and fusion proteins

The following monoclonal antibodies against CTLA-4 (CD152) were used in these studies: BNI3 (BD Pharmingen, SanDiego, CA), AS32 and AS33 (Antibody Solutions, Palo Alto, CA), are monoclonal antibodies that recognize extracellular epitopes in the CTLA-4 molecule. The MOPC-21C antibody (Sigma-Aldrich, St. Louis, MO) was used as a negative control. ELISA assays for CTLA-4 were done as described previously [25]. B7.1-Ig (CD-80) and B7.2-Ig (CD86) fusion proteins (R&D Systems, Minneapolis, MN) were biotinylated with the use of a commercially available kit (Pierce Immunochemical, Rockford, IL). The Muc18-Ig protein (Muc-Ig) fusion protein was produced by transfecting CHO cells with a commercially available plasmid (Novagen) encoding a Muc18-Ig fusion as described [15,29].

### Purification of blood-derived sCTLA-4

Plasma samples used in these studies were obtained from humans undergoing therapeutic plasmapheresis for myasthenia gravis. Documented informed consent was obtained for each of the subjects enrolled in this study. This study was performed under the oversight of the Institutional Review Board of Aurora Health Care (Protocol # L-04-35E). Plasma was frozen at -20°C until use. 200 mls of plasma were diluted with 20 mM Tris-HCl in water until the pH of the solution reached 6.8. Typically this required a mixture 1 volume of plasma to 9 volumes of 20 mM Tris-HCl. This mixture was then passed through a column containing 300 mls of Q sepharose (GE healthcare). Flow through was collected and solid ammonium sulfate was added to create a 45% saturated solution. After overnight incubation at 4°C, precipitate was collected by centrifugation at 1,800 g for 15 minutes. The pelleted material was resuspended in 20 mls of phosphate buffered saline (PBS, pH 7.4) and dialyzed using a 1 kDa molecular weight dialysis tubing in PBS with 2 buffer exchanges. This material was passed over a 2 ml column, linked to the MOPC-21 monoclonal antibody using the Pierce Aminolink Plus Immobilization kit, and flow through was then passed over a column containing the antibody AS32. Material was eluted from the AS32 column using elution buffer (Pierce) and collected in 1 ml fractions.

### Blood-derived sCTLA-4 binding to B7.1-Ig and B7.2-Ig

96 well ELISA plates were coated with 100 ul of fractions collected from the AS32 column overnight at 4°C. After coating, plates were washed and incubated with 250 μl block solution (PBS containing 1% w/v bovine serum albumin and 5% w/v sucrose) for 1 hour at room temperature to block nonspecific protein binding sites. Blocked wells were incubated with the biotinylated proteins B7.1-Ig, B7.2-Ig, Muc18-Ig, or AS33 at room temperature for 1 hour. Next, wells were washed, incubated with streptavidin HRP (Zymed Carlsbad, CA) for 20 minutes and washed again. TMB (Pierce Immunochemicals) was added to the wells, the HRP reaction proceeded for approximately 15 minutes, was stopped by addition of 1N H2SO4, and absorbance was read during illumination with light at 450 nm.

### Protein A binding of sCTLA-4

Recombinant sCTLA-4, produced as described [22], or plasma diluted 1:10 with 150 mM NaCl and 10 mM Tris pH 7.4 (TBS), was passed over a column containing 1 ml of protein A sepharose (Pierce). Flow through was collected in 1 ml fractions. 10 mls of TBS was passed over the column to wash unbound material and was collected in 1 ml fractions. Bound material was eluted from the column in 100 mM glycine pH 3, collected in 1 ml fractions and neutralized with 100 ul of 10xTBS. Pre-column, flow through, wash, and eluted fractions were tested in ELISA for the presence of sCTLA-4.

### Gel filtration

1.5 mls of serum was diluted with 1.5 mls of PBS containing recombinant sCTLA-4, and passed through a column (1.5 cm diameter) containing approximately 150 ml of sephacryl S200 (GE healthcare). The first 4 mls were discarded and then 2 ml fractions were collected by hand. Each fraction was tested for the presence of blood-derived sCTLA-4 or recombinant sCTLA-4 using elisa assays.

### Protein Identification

Identification of proteins from 1D-SDS PAGE was performed by Proteomic Research Services (. Ann Arbor, MI). Proteins that eluted from the AS32 column were precipitated in 70% ethanol and pelleted by centrifugation 14,000 rpm in a microcentrifuge for 5 minutes. The pellet was dissolved at room temperature in 5% SDS, 10 M Urea, 10 mM Tris buffer (pH 6.8), and 10% 2-mercapto ethanol, and separated using NuPage 12% bis-tris SDS PAGE gels in MES running buffer (Invitrogen, Carlsbad, CA) and visualized by staining gels with coomassie blue. Proteins were reduced, alykylated and trypsin digested in isolated gel fragments, and were identified using LC/MS/MS.

Protein identification by 2D-DIGE was performed by Applied Biomics (. Hayward, CA). Purified proteins were labeled with either Cy3 or Cy5. The first dimension separation consisted of isoelectric focusing over pH 3-10. The second dimension separation was carried out using 8-14% gradient SDS-PAGE. Differentially expressed proteins were cut out and digested with trypsin before analysis with mass spectroscopy.

## Results

### The CTLA-4-specific monoclonal antibodyAS32 isolates B7-binding proteins from blood

**Figure 1:**
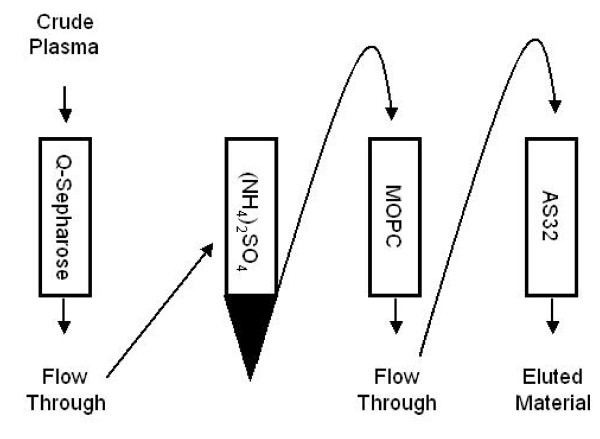
**Schema for isolating sCTLA-4 from human blood**. 1) Crude plasma was passed over a Q sepharose column to remove albumin and other contaminants. 2) Material that did not bind Q sepharose was concentrated by ammonium sulfate precipitation. 3) Concentrated material was passed over an antibody column containing an irrelevant monoclonal antibody against MOPC. 4) MOPC column flow through was passed over a column containing the CTLA-4 specific antibody AS32. Bound material was then eluted and collected.

In order to provide a more detailed biochemical characterization of sCTLA-4 from human plasma, we used the purification scheme depicted in Figure 1 with specific details given in the Methods section. The purification scheme required large quantities of human plasma. Therefore, we collected discarded plasma from patients receiving plasmapheresis as part of a therapy to treat myasthenia gravis. This provided a safe means of obtaining large volumes of plasma from humans. We collected samples from three patients that tested positive in CTLA-4 ELISA assays and one patient that tested negative. The plasma was used to perform biochemical analyses of CTLA-4 immunoreactive material and no relationship to disease was studied.

**Figure 2:**
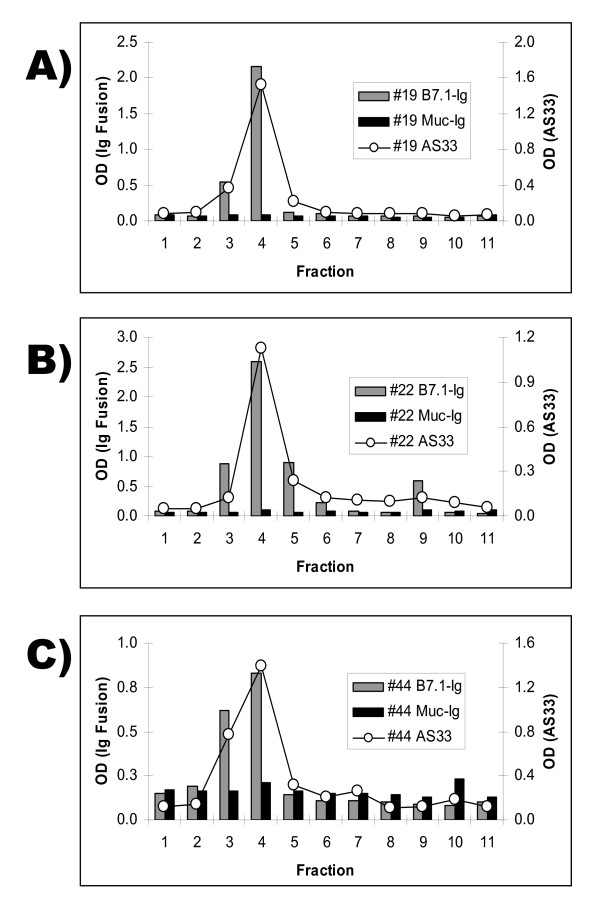
**Molecules that bind B7.1 can be isolated from plasma using CTLA-4 specific antibodies**. CTLA-4 positive plasma, from subjects 19 (A), 22 (B), and 44 (C), were passed through a column linked to the CTLA-4 specific antibody AS32. Fractions, containing material that eluted from the column at low pH, were bound to 96 well plates and tested for interaction with various biotinylated proteins by ELISA: B7.1-Ig (gray bars), Muc-Ig (black bars), and the AS33 antibody (circles).

**Figure 3:**
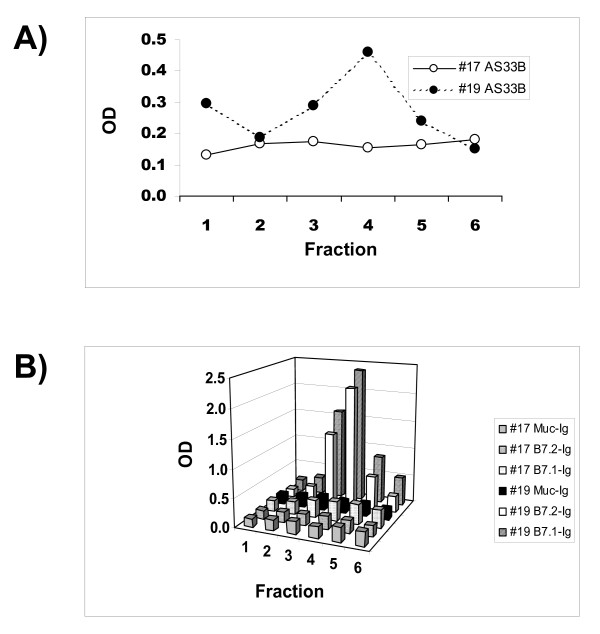
**Material that binds B7.1 and B7.2 can only be isolated from blood that contains molecules recognized by CTLA-4 specific antibodies**. Plasma from subjects 17 (CTLA-4 negative) and 19 (CTLA-4 positive) were passed over a column linked to the CTLA-4 specific antibody AS32. Material that eluted from the column under stringent wash conditions was collected in fractions, and was used to coat the wells of a 96 well plate. In panel (A) these wells were probed with the CTLA-4 specific antibody AS33. Panel (B) shows the binding of B7.1-Ig, B7.2-Ig, and Muc-Ig to the coated wells.

Eluted material was collected in fractions and tested in sandwich ELISA assays for CTLA-4 epitopes and for functional activity in a B7 binding assay. Figure 2 shows the results of experiments from three distinct plasma samples. Peak reactivity with CTLA-4 specific antibodies correlates with binding to the B7.1 protein. The specificity of the binding to B7.1 was verified when the isolated material did not bind to an irrelevant fusion protein (Muc-Ig). Similar results were also obtained with the B7.2-Ig fusion protein (Figure 3), demonstrating that material eluted from anti-CTLA-4 affinity columns binds to CTLA-4 ligands. When the plasma samples lacking CTLA-4 immunoreactive material were subjected to an identical affinity purification procedure, eluted material displayed minimal reactivity in CTLA-4 ELISA and did not bind either B7.1 or B7.2 (Figure 3).

### Identification of proteins enriched by an AS32 column

**Figure 4:**
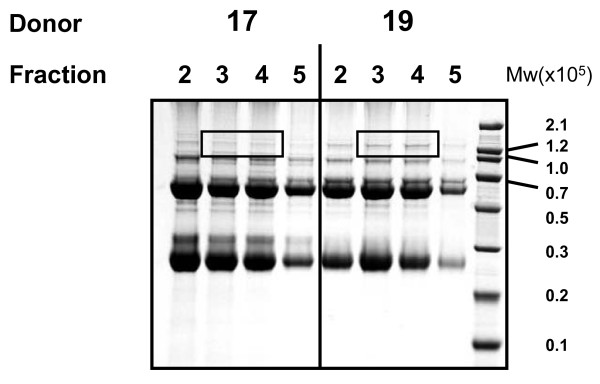
**1D SDS-PAGE analysis of material captured by a CTLA-4 specific affinity column**. CTLA-4 negative plasma (donor 17) and CTLA-4 positive plasma (donor 19) were passed through a column linked to the CTLA-4 specific antibody AS32. Material was eluted at low pH and collected into fractions that were separated by 1D-SDS PAGE. Coomassie blue staining was used to visualize protein patterns. A box marks the single band enriched in donor 19 compared to donor 17.

Proteins eluted from anti-CTLA-4 affinity columns, were separated by one-dimensional SDS-PAGE and visualized with coomassie blue staining. Figure 4 shows a representative experiment comparing proteins isolated from plasma that either lacked or contained anti-CTLA-4 immunoreactive material. The ~ 150 kDa band marked by a box is enriched in anti-CTLA-4 reactive samples from 3 different donors (not shown) compared to the anti-CTLA-4 negative sample. Proteins in the highlighted gel fragment were identified by LC/MS/MS and are listed in Table 1. Protein sequencing was performed on material isolated from 2 different positive donors. A light chain from a hepatitis B specific antibody was the only protein common to both samples.

**Table 1:** LC/MS/MS identification of proteins in the 150 kd band unique to subjects testing positive in ELISA using CTLA4 specific antibodies.

**Subject**	**Protein ID**	**Accession Number**	**% Sequence Coverage**
19	Chain A, Human Factor Vii C2 domain	**GI |15825659**	32.7
19	Protein NIG51 lambda Bence-Jones	**GI |223229**	30.6
19	Ig heavy chain V-III region CAM	**GI |123847**	38.5
19	Immunoglobulin heavy chain [homo sapiens]	**GI |10334553**	39.8
19	Unnamed protein product	**GI |28317**	11.3
19	Anti HBs antibody light-chain Fab fragment [homo sapiens]	**GI |11275330**	35.0
19	Unnamed protein product [homo sapiens]	**GI |34527640**	33.4
19	Keratin 1 [homo sapiens]	**GI |17318569**	15.7
44	Ig A L	**GI |229536**	21.8
44	Anti HBs antibody light-chain Fab fragment [homo sapiens]	**GI |11275330**	26.3
44	IGHG1 protein [homo sapiens]	**GI |50925957**	29.4
44	Keratin 1	**GI |17318569**	8.7
44	IGHM protein [homo sapiens]	**GI |33873884**	7.3
44	Keratin type I cytoskeletal 9 [homo sapiens]	**GI |81175178**	8.7

### Recombinant sCTLA-4 and anti-CTLA-4 affinity-purified material from human plasma differentially bind protein A and have different molecular masses

**Figure 5:**
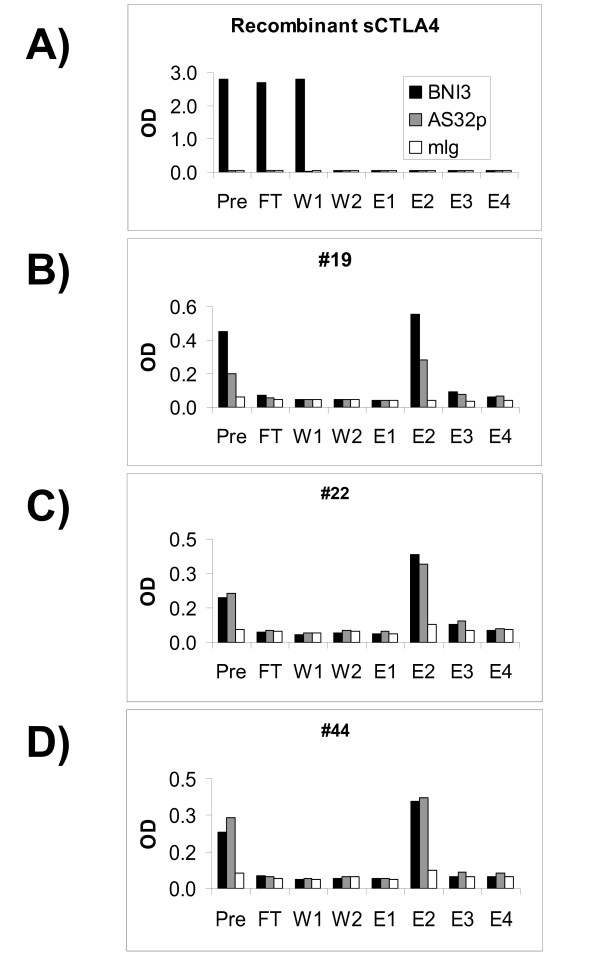
**Protein A binds blood-derived molecules that contain CTLA-4 epitopes, but does not interact with recombinant sCTLA-4**. Recombinant CTLA-4 (A) and plasma from CTLA-4 ELISA positive donors 19 (B), 22 (C) and 44 (D) were passed over protein A columns. Pre-protein A (Pre), protein A flow through (FT), washes (W1 and W2), and fractions eluted at low pH (E1, E2, E3, and E4) were analyzed by ELISA. Black bars represent BNI3 (coat)/AS33B (capture), grey bars represent AS32 (coat)/AS33B (capture), and white bars anti-MOPC (coat)/AS33B (capture).

In an effort to remove contaminating immunoglobulin from the molecules recognized by anti-CTLA-4 antibodies, we passed plasma through columns linked to protein A. Unexpectedly, CTLA-4 immunoreactive material from plasma bound to the protein A column and was only released at low pH (5B-D). In comparison, recombinant sCTLA-4 that passed over protein A columns was present in flow- through and in the neutral pH column wash solution, but it did not elute at low pH (Figure 5A). This indicates that the CTLA-4 immunoreactive material in blood exhibits immunoglobulin like properties in contrast to recombinant sCTLA-4.

**Figure 6:**
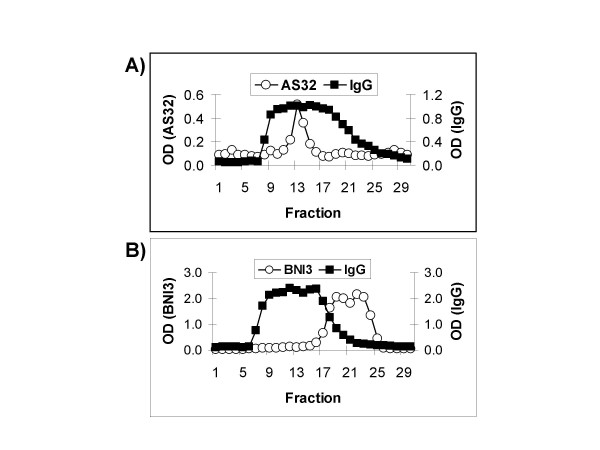
**Size exclusion chromatography separates recombinant sCTLA-4 from blood-derived CTLA-4 immunoreactive material**. A) Following gel filtration, fractions of CTLA-4 positive serum were analyzed with the sCTLA-4 specific antibodies AS32 (coating antibody) and AS33 (reporter antibody) and with antibodies specific for human IgG. B) Anti-CTLA-4 (BNI3 coat/AS33 reporter) ELISA and anti-human IgG ELISA were used to analyze fractions of CTLA-4 negative serum mixed with recombinant sCTLA-4 after separation by gel filtration. Circles represent CTLA-4 ELISA, and squares represent human IgG ELISA.

In addition, the finding that a CTLA-4-specific affinity column enriched a 150 kDa plasma protein (Figure 4) was surprising given the primary sequence of sCTLA-4 predicts its mass to be 23 kDa. In an effort to determine the molecular mass of CTLA-4 immunoreactive material by a second method, we performed gel filtration on serum and tested fractions in ELISA assays using CTLA-4 specific antibodies. Human IgG served as an internal size standard. Blood-derived CTLA-4 immunoreactive material co-eluted with the bulk of IgG indicating that it has a molecular mass of approximately 150 kD (Figure 6A). This result was repeated in samples obtained from 5 other donors whose blood reacts with CTLA-4 specific antibodies (not shown).

For comparison, serum from a donor lacking molecules recognized by CTLA-4 specific antibodies was spiked with recombinant sCTLA-4 and then passed over a sizing column. Recombinant sCTLA-4 clearly eluted from the column after IgG (Figure 6B). The minimal overlap of the IgG fractions with fractions containing recombinant sCTLA-4 demonstrates that recombinant sCTLA-4 is smaller than 150 kD.

### 2D gel electrophoresis and mass spectroscopy of proteins enriched on an anti-CTLA-4 affinity column

Because molecules purified by anti-CTLA-4 affinity chromatography exhibit biochemical properties similar to human IgG, we attempted to separate this material from IgG using 2 dimensional gel electrophoresis that separates molecules on the basis of isoelectric point and size. For comparison, molecules that bound to a column containing an immobilized irrelevant antibody (MOPC), and molecules bound to the AS32 column, were labeled with different color fluorescent molecules and separated on the same gel. The fluorescent dyes allowed identification of proteins that were enriched by the AS32 column. 17 spots, which were at least 2-fold more abundant in the AS32 eluted material versus the MOPC material, were analyzed by mass spectroscopy, and their identity is shown in Table 2. There were no common proteins identified in this analysis when compared to mass spectroscopy performed on bands identified on SDS-PAGE gels (Figure 4 and Table 1). Interestingly, we found another light chain from an antibody specific for hepatitis B surface antigen. The anti-Hepatitis B surface antigen light chains identified by the 1 dimensional and 2 dimensional protocols are distinct light chains as they only share 30% sequence similarity (not shown).

**Table 2:** Mass Spectroscopy Identification of Proteins Enriched by Affinity Purification of Plasma with a CTLA4 Affinity Column and Analyzed by 2D-DIGE

**Spot**	**Fold Increase As32:MOPC**	**Protein Name**	**Accession No**.
1	3.2	Plasminogen	**GI |38051823**
2	1.9	Plasminogen	**GI |38051823**
3	2.8	Plasminogen	**GI |38051823**
4	3.5	Immunoglobulin heavy chain	**GI |10334573**
5	4.8	Immunoglobulin heavy chain	**GI |10334573**
6	2.1	hypothetical protein	**GI |34365168**
7	1.9	hypothetical protein	**GI |34365168**
8	5.7	This CDS feature is included	**GI 1684931**
9	6.2	complement C1q subcomponent, α	**GI |54781353**
10	5.0	complement C1q subcomponent, α	**GI |54781353**
11	8.2	immunoglobulin lambda light chain C region	**GI |386813**
12	2.4	Unknown (protein for MGC:31941)	**GI |18044245**
13	5.4	Chain B, Globular Head Of The Complement System	**GI |38492827**
14	2.2	anti HBs antibody light-chain Fab	**GI |11275326**
15	2.4	α-Entamoeba histolytica immunoglobulin κ light chain	**GI |5360675**
16	3.2	IGLC1 protein	**GI |20380868**
17	2.2	anti HBs antibody light-chain Fab	**GI |11275326**

Plasma proteins that preferentially bound a to the CTLA4 specific antibody AS32 compared to the irrelevant control antibody MOPC were identified by 2D-DIGE. Spots were picked and identified by MALDI spectroscopy.

## Discussion

Our biochemical analyses demonstrate that the molecules in human blood that are recognized by anti-CTLA-4 antibodies are not a simple product of the sCTLA-4 alternate transcript. We previously described sCTLA-4 immunoreactive material in blood plasma from patients with autoimmune thyroid disease [22]. In that study, Western blotting of material from immunoprecipitates, using a pool of commercially available monoclonal antibodies to CTLA-4, identified proteins with a molecular mass of 23 kDa consistent with the size of recombinant sCTLA-4. We also noted the observation of larger material (> 100 kDa) in those immunopreciptates but did not characterize this species further. The CTLA-4 immunoreactive material described in this communication may be the larger molecules noted in our previous studies.

This study identifies material that reacts with multiple anti-CTLA-4 antibodies and with known ligands of CTLA-4, B7.1 (CD80) and B7.2 (CD86). Additionally, its molecular size is approximately 150 kDa, it binds protein A with high affinity, and when analyzed by mass spectroscopy, we find human antibodies. Despite the fact that the molecules studied exhibit characteristics of CTLA-4-Ig fusion proteins, none of our plasma donors received exogenous CTLA-4-Ig (Orencia) as a therapy. Therefore the isolated molecules are endogenous to our plasma donors.

Though we did not conclusively identify these molecules, we found 2 separate light chains that originated from human immunoglobulins specific for Hepatitis B surface antigen (Tables 1 and 2). One of the Hepatitis B specific light chains was found in 2 different subjects, and both light chains were isolated from a single donor. While it is possible that we have identified an idiotype network involving epitopes on the Hepatitis B surface antigen, CTLA-4, and some immunoglobulins, comparing the sequences with the bl2seq program on the NCBI website  did not reveal any obvious sequence homology among these molecules. A more detailed structural analysis is warranted.

**Figure 7:**
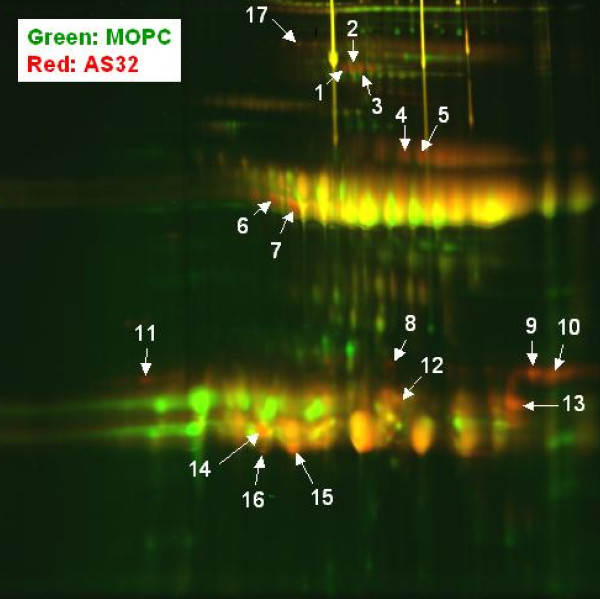
**2 dimensional DIGE comparison of plasma proteins that bind to either the CTLA-4 specific antibody AS32 or to an anti-MOPC isotype control antibody**. Plasma containing molecules that react with the antibody AS32, as determined by ELISA, was passed over a column containing an anti-MOPC antibody. Flow through of that column was passed over a column linked to the antibody AS32. Both columns were washed in PBS, and bound material was eluted at low pH. Eluted fractions containing peak material were concentrated by binding to protein A beads and analyzed by 2 dimensional DIGE. Material that eluted from the anti-MOPC column was labeled with a green dye, and material that eluted from the AS32 column was labeled with a red dye. Yellow spots correspond to areas of the gel containing the same amount of signal from red and green labeled proteins.

Perhaps most importantly, our findings highlight the fact that the sCTLA-4 measured in human plasma by studies of autoimmune disease does not represent a simple gene product of the sCTLA-4 transcript. Though the isolated material was recognized by several CTLA-4 specific antibodies and interacted with B7.1 and B7.2 proteins, mass spectroscopy analysis found no evidence of the CTLA-4 protein in the isolated material. It is possible that CTLA-4 protein escaped our mass spectroscopy analysis. As shown in figure 4, only a 150 kDa protein was more abundant in samples that are positive by CTLA-4 ELISA than in the negative sample. sCTLA-4, having a mass of 23 kDa, may be present in the very abundant band at approximately 23 kDa, but there was no evidence that this band was enriched during our isolation procedure. Because of the abundant material at ~ 23 kDa and ~ 50 kDa in both the positive and negative samples we realized it was possible that less abundant proteins may be obscured if they comigrated with more abundant proteins. Therefore, we chose to analyze the samples by 2D-DIGE in an effort to resolve sCTLA-4 from contaminating abundant proteins. Computer analysis identified the proteins most highly enriched by the CTLA-4 specific affinity column, and again sCTLA-4 was not found even when proteins of approximately 23 kDa were analyzed (figure 7 and table 2).

Our gel filtration and protein A binding data suggest that if sCTLA-4 exists in the blood samples we analyzed, it appears to be complexed with immunoglobulin. Another possibility is that blood contains protein(s), either immunoglobulins or a novel protein associated with immunoglobulins, that interact with the ligands of CTLA-4 and with CTLA-4 specific antibodies.

We believe the presence of CTLA-4 immunoreactivity in plasma is not likely due to heterophile antibodies that are known to interfere with two site immunoassays [30,31]. The samples selected for analysis in these studies were screened to be negative for binding to irrelevant mouse IgG. Furthermore, the Muc-Ig negative control used in the B7 binding assays contained the identical Ig fusion partner as the B7-Ig proteins. Finally, if present, heterophile antibodies should have been removed by the MOPC column during the isolation procedure.

Our findings may reconcile the apparent discrepancy between reports of elevated levels of sCTLA-4 in plasma from patients with autoimmune disease and the report of decreased levels of the sCTLA-4 transcript among individuals with the CT60 allele of the CTLA-4 gene. CT60 is a single nucleotide polymorphism within the CTLA-4 locus [18] and the "G" allele is associated with both susceptibility to Type 1 diabetes and low levels of the sCTLA-4 transcript. Because sCTLA-4 in blood is probably not the direct product of the sCTLA-4 transcript, the lack of correlation between transcript and plasma protein, is not surprising. In addition, immunoassays designed to measure sCTLA-4 levels may not reliably quantify blood-derived sCTLA-4 because of its uncertain identity. In this regard, it is important to interpret reports of sCTLA-4 levels with caution, and to re-examine the possible relationship of circulating CTLA-4 levels and human disease. To this point, we have recently reported a lack of association between levels of sCTLA-4 protein in blood and several of the common polymorphisms that show population genetic associations with a variety of autoimmune disease [32].

## Conclusion

Molecules from human blood that have been labeled sCTLA-4 are not simply a direct product of an alternatively spliced transcript of the CTLA-4 gene. These molecules exhibit properties of CTLA-4 and of immunoglobulins. Though some studies have found a correlation between circulating levels of the putative sCTLA-4 molecule and the presence of autoimmunity, this relationship is poorly understood. This study improves understanding of the biochemical nature of what has been called sCTLA-4 and will help subsequent analyses to elucidate the role of this molecule in autoimmune disorders.

## Competing interests

The authors declare that they have no competing interests.

## Authors' contributions

MT and MKO conceived of and designed the study, drafted the manuscript and performed immunoassays and biochemical analyses and helped to draft the manuscript. KK and KD carried out immunoassays and biochemical analyses. BOK participated in design of the study and in its coordination and helped to draft the manuscript. All authors read and approved the final manuscript.
